# Effective identification of varieties by nucleotide polymorphisms and its application for essentially derived variety identification in rice

**DOI:** 10.1186/s12859-022-04562-9

**Published:** 2022-01-10

**Authors:** Xiong Yuan, Zirong Li, Liwen Xiong, Sufeng Song, Xingfei Zheng, Zhonghai Tang, Zheming Yuan, Lanzhi Li

**Affiliations:** 1 Hunan Engineering and Technology Research Center for Agricultural Big Data Analysis and Decision-Making, Hunan Agricultural University, Changsha, 410128 China; 2 State Key Laboratory of Hybrid Rice, Hunan Hybrid Rice Research Center, Changsha, 410125 China; 3 Hubei Key Laboratory of Food Crop Germplasm and Genetic Improvement, Food Crop Institute, Hubei Academy of Agricultural Sciences, Wuhan, 430064 China; 4 College of Food Science and Technology, Hunan Agricultural University, Changsha, 410128 China

**Keywords:** Variety identification, Essentially derived variety, Rice, SNP, Whole-genome sequencing

## Abstract

**Background:**

Plant variety identification is the one most important of agricultural systems. Development of DNA marker profiles of released varieties to compare with candidate variety or future variety is required. However, strictly speaking, scientists did not use most existing variety identification techniques for “identification” but for “distinction of a limited number of cultivars,” of which generalization ability always not be well estimated. Because many varieties have similar genetic backgrounds, even some essentially derived varieties (EDVs) are involved, which brings difficulties for identification and breeding progress. A fast, accurate variety identification method, which also has good performance on EDV determination, needs to be developed.

**Results:**

In this study, with the strategy of “Divide and Conquer,” a variety identification method Conditional Random Selection (CRS) method based on SNP of the whole genome of 3024 rice varieties was developed and be applied in essentially derived variety (EDV) identification of rice. CRS is a fast, efficient, and automated variety identification method. Meanwhile, in practical, with the optimal threshold of identity score searched in this study, the set of SNP (including 390 SNPs) showed optimal performance on EDV and non-EDV identification in two independent testing datasets.

**Conclusion:**

This approach first selected a minimal set of SNPs to discriminate non-EDVs in the 3000 Rice Genome Project, then united several simplified SNP sets to improve its generalization ability for EDV and non-EDV identification in testing datasets. The results suggested that the CRS method outperformed traditional feature selection methods. Furthermore, it provides a new way to screen out core SNP loci from the whole genome for DNA fingerprinting of crop varieties and be useful for crop breeding.

**Supplementary Information:**

The online version contains supplementary material available at 10.1186/s12859-022-04562-9.

## Background

Fast, accurate, and efficient varieties or cultivars identification and characterization are essential for crop varieties' breeding, registration process, seed production, trade, inspection, and patents protection [1]. As a staple food, rice is consumed by more than 3.5 billion people worldwide. Therefore, breeders must continuously develop high-yield and elite-quality rice varieties to meet the demands of the increasing population and food consumption. Identifying, screening, and utilizing the rice germplasm resources are the first steps of rice introduction and improvement programs. At present, more than 780,000 rice germplasm are available in gene banks worldwide, theoretically retaining all the gene resources of rice [2, 3]. However, many varieties have similar genetic backgrounds, even some essentially derived varieties (EDVs) involved, resulting in difficulties for identification and breeding progress [4, 5]. Thus, it is urgent to establish a varieties fingerprint map based on a sufficient number of varieties in the germplasm resource, assess variety distinctness as we can [6], and especially apply it for EDV identification for prompting crops breeding [7]. Only through this way can the genetic relationship among varieties be effectively analyzed and then effectively guide the breeding parents' selection, providing valuable information for further rice breeding [8, 9].

The traditional species or variety identification approach involves observing and recording morphological characters [1, 6]. Since morphological characters are influenced by the environment and not available at all growth stages, the traditional approach is not practical for rapidly separating extensive collections. It also has compromised precision and is time-consuming. The use of molecular markers is a modern and suitable approach to cultivar and variety identification [10]. Molecular markers can save the time of routine field investigation and data collection and have the advantages of being unaffected by the environment and extremely rich in variation. Moreover, it is especially suitable for closely related varieties identification [11]. SSR and SNP markers were identified as the most widely used marker system for plant variety characterization for stability and effectiveness and recommended their use as an additional marker system in conjunction with morphological characters by the International Union for the Protection of New Varieties of Plants (UPOV) [12, 13]. Generally, a SSR contains more polymorphic information content (PIC) than does a SNP since SSRs are often multi-allelic while SNPs are mostly bi-allelic. However, the nomenclature of SNP is much simpler than that of SSR, which makes the analysis and sharing of results much more accessible [14]. Furthermore, SNPs are more abundant and stable in genomes than SSRs and evenly distributed in a whole genome, while lots of SSRs trends located in the non-coding region of genes [15, 16].

In 2010, Jung et al. [17] first reported the development of a panel of SNP markers for variety identification in peppers. With 40 SNPs, they could discriminate 97.5% of the 81 commercial hot cultivars and 100% of the 17 sweet pepper cultivars. Cabezast et al. [18] selected a set of 48 stable SNP markers with a high discrimination power and a uniform genome distribution, which was proposed as a standard set for grapevine genotyping. Hinze et al. [19] analyzed the diversity of cotton (*Gossypium hirsutum* L.) germplasm using the CottonSNP63K Array and screened out SNPs to efficiently discern differences among cultivars. In 2018, fifty core markers from 2.54 million SNPs obtained by aligning resequencing data of cabbage inbred lines were selected and used to establish a DNA fingerprint database of 59 cabbage varieties. An artificially mixed population validated the core SNP markers. The SNP fingerprinting constructed with core markers could identify the cabbage variety's distinctness and authenticity [20]. Almost of previous studies of varieties identification ordered SNPs with PIC at first, then manually screened out the SNPs with high PIC that evenly distributed on the chromosomes and constructed a fingerprint map [10, 15]. The manual screening process often was not easy to repeat. Meanwhile, when the sample size was large, selecting an optimal SNP combination set was hard to distinguish all the varieties in a specific study with this strategy. This dilemma is possible due to the inefficient screening approaches and/or limitation of polymorphic markers.

Recently, with the development of sequencing technology, high-throughput SNPs have been widely generated in lots of crops [21]. Zhang et al. [22] developed target SNP-seq and established a DNA fingerprint of 261 cucumber varieties by target SNP-seq with 163 perfect SNPs from 4,612,350 SNPs based on 182 cucumber resequencing datasets. In 2018, the 3000 Rice Genome Project (3 K RGP) had publicly released the sequence data of 3024 rice germplasm from the 780,000 rice materials in the global rice germplasm library, with an average sequencing depth of 14×. It shared more than 3 million SNP markers, providing a great genetic resource for identifying rice varieties [2, 3]. However, to save cost, selecting a minimal set of features from enormous SNP markers to accurately identify varieties is a significant problem in constructing a rice fingerprint map. Besides that, scientists should take EDVs and non-EDVs (or distinct varieties) discrimination account into varieties identification research for the EDVs impacted breeding innovation negatively [8, 22, 23].

The objectives of this study were to (1) develop a fast, cost-effective screening SNP markers procedure for varieties identification without manual operation process; (2) construct a rice fingerprint map and apply it for EDV and non-EDV discrimination with independent test datasets; (3) code the method with R language for easy application.

## Materials and methods

### Genotype dataset of materials

There are three rice SNP genotype datasets involved in this study. We downloaded the first dataset from the 3000 Rice Genome Project (https://snp-seek.irri.org/), including ~ 4.04 million core SNPs of 3024 rice accessions from 89 different countries or regions [2]. The core SNPs passed quality control were maintained by removing SNPs with > 20% missing calls and MAF < 1% and using a two-step linkage disequilibrium pruning procedure with PLINK (version 1.9) [24]. Then SNP genotype imputation was performed with beagle software (version 5.0) [25]. The genome sequences of the 3,024 accessions represent various varietal types of diverse origins and the availability of additional high-quality rice reference genomes. Rice gene annotation information was obtained from the Genome database of NCBI GenBank (http://www.ncbi.nlm.nih.gov/, update to Nov. 6th, 2020). Then 99,253 SNP markers located in the genes' coding region (cSNPs) were extracted and used following rice fingerprint mapping modeling. The training dataset contains 3024 varieties with 99,253 SNPs, denoted as Train_Data (99,253 × 3024).

The second dataset contains 1568 rice varieties genotyped using a genome-wide high-density rice array (HDRA). Wang et al. [2] imputed the SNP genotype of these different 1568 rice varieties with above 3024 rice genomes as reference. We downloaded the imputed SNP genotype dataset with 404 k core SNPs from the 3000 Rice Genome Project (https://snp-seek.irri.org/). The same 99, 253 cSNPs as in Train_Data were extracted to form the first independent test dataset, denoted as Test_Data1 (99,253 × 1568).

The third dataset contains 401 rice varieties. Their seeds were obtained from the China rice breeders and planted in Wuhan in 2013. On average, these materials were sequenced on the Illumina HiSeq2500 platform at 11 × genome coverage. By quality control, 1,894,012 high quality SNPs with minor allele frequency (MAF) > 5% and missing rate < 20% were obtained. Among them, there are 319,624 SNPs also detected in 3K-RGP, respectively. The 85, 860 cSNPs that were also identified in Train_Data were extracted to form the second independent test dataset, denoted as Test_Data2 (85, 860 × 401).

**Fig. 1:**
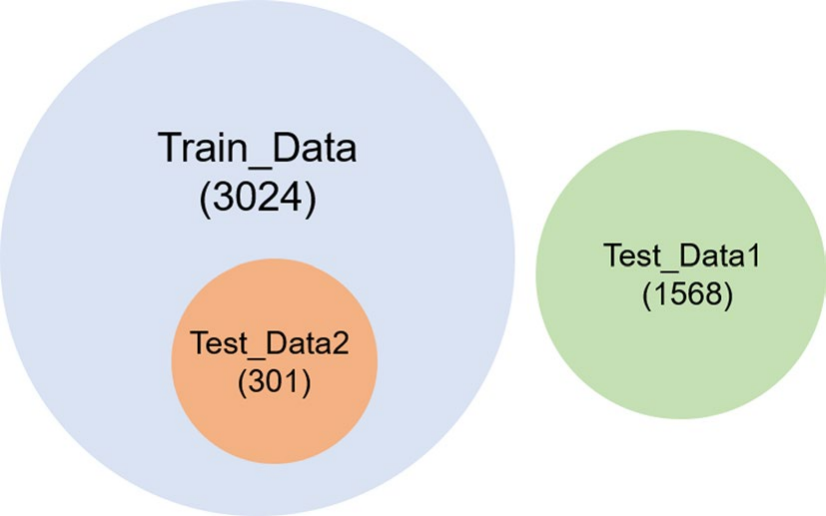
Venn diagram of the composition of the training dataset and two independent test datasets

The composition of the training dataset and two independent test datasets are shown in Fig. 1. The varieties in Test_Data1 are different from those in Train_Data, denoted as external varieties. The varieties in Test_Data2 were collected from some breeding experts and have the same variety name as some varieties in Train_Data, denoted as internal varieties.

### Marker polymorphism analysis

The software R language was used to calculate each cSNP marker's polymorphism information content (PIC) in the Train_Data. The calculation formula is

1 $$PIC=1-\sum {f}_{i}$$

where fi is the genotype frequency of the given SNP site i [26, 27].

### Genomic similarity testing of varieties in Train_Data

Identity scores (IS) of SNPs were used to calculate the genomic similarity of varieties [28], which assists in discriminating essentially derived variety (EDV) from non-essentially derived variety (non-EDV) [23]. The IS was the total accumulated score within a 20-kb window divided by the total number of SNPs. The formula of IS was as below:

2 $$IS = \frac{{\sum\limits_{i}^{N} {(1 - \left| {Ds_{1i} - Ds_{2i} } \right|)} }}{{\text{number of SNPs in the window}}}$$

In this formula, Dsi refers to the distance value of sample allele to the reference allele at a given SNP site i; Dsi is 0, 0.5, and 1, separately for the homozygous allele that same as the reference allele, heterozygous allele, and homozygous allele that different from the reference allele at given SNP site i. N is the total number of SNPs within a 20-kb window. In this study, taking Oryza Sativa L. cv. Nipponbare as reference genome, IS between every two varieties in the Train_Data were calculated with the 404 k core SNPs. According to Longping Yuan's suggestion, IS = 97.5% was set as the threshold for EDVs' determination of rice varieties with SNPs on the whole genome [23]. If two or more varieties are regarded as EDVs in Train_Data, only one is randomly retained for following distinct varieties analysis.

### Selecting SNPs from the training dataset to construct fingerprint map

#### Concept of conditional random selection (CRS) method

**Fig. 2:**
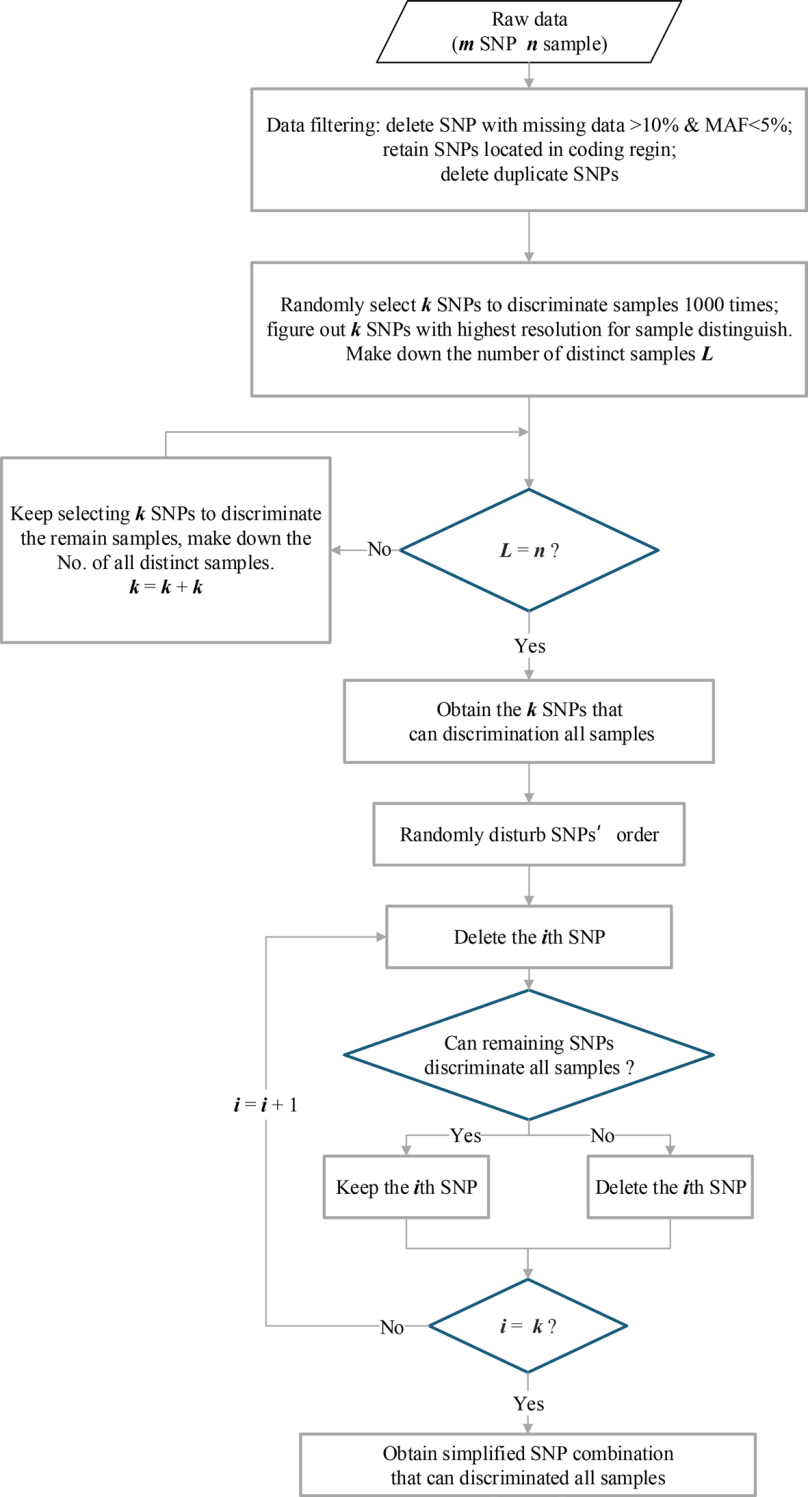
Flow chart of the Conditional-Random-Selecting SNP method

**Fig. 3:**
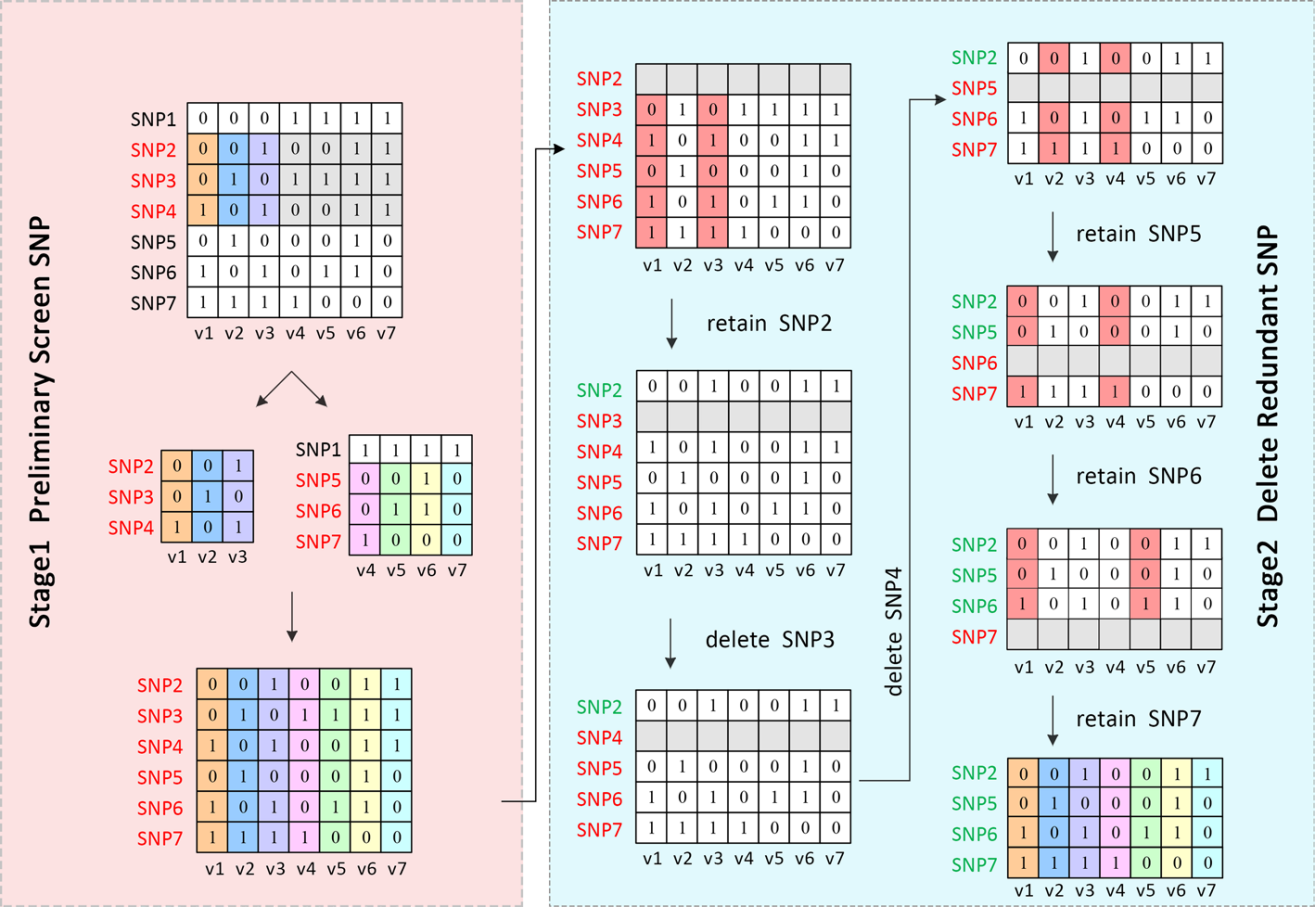
Schematic of the two stages of the Conditional-Random-Selecting SNP workflow

This study proposed a new method, Conditional Random Selection (CRS), for fast and accurate identifying varieties. The flowchart of the CRS method was shown in Fig. 2. To explain the concept of the CRS method clearly, we took the dataset with seven varieties (V1–V7) and seven SNPs as an example with a schematic diagram (Fig. 3).

Stage 1: SNPs Preliminary screening. At first, randomly selected three SNPs to separate accessions by constructing specific haplotypes. Assuming that SNP2, SNP3 and SNP4 were selected in the first round. The genotype combinations of the three SNPs were different from each other among varieties V1(0, 0, 1), V2(0, 1, 0), and V3(1, 0, 1), which denoted as specific haplotypes. In contrast, the situation is different in V4–V7. The three SNPs' genotype combination for V4 and V5 was (0, 1, 0), and for V6 and V7 were (1, 1, 1), referred to as a non-specific haplotype (Fig. 3). Then the specific haplotype of the remaining varieties (V4–V7) was constructed by randomly selecting three SNPs from the remaining markers (SNP1, SNP5–SNP7), repeated this procedure until all varieties had specific haplotypes.

Stage 2: Redundant SNP deletion. Randomly shielded one SNP at a time, if the remaining SNPs combination can still discriminate all varieties from each other, indicating the shielded SNP was a redundant one. We filtered out this redundant SNP. Otherwise, kept the SNP. We repeated this step several times until all the marks were checked. As shown in Fig. 3, SNP 3, 4, and 7 are redundant SNPs. The haplotype constructed by the genotype combination of SNP2, SNP5, and SNP6 can still distinguish all seven varieties, forming a simplified SNP combination.

#### Varieties identification in training set by using the CRS method

Compared with the 404 k SNPs covering the whole genome of 3024 varieties in Train_Data, 99,253 cSNPs located in the gene coding region are significantly reduced. To make the selected cSNPs for varieties identification in Train_Data also be able to identify EDVs effectively, we prefer to choose cSNPs that be polymorphic in EDVs for other distinct varieties (non-EDVs) identification with the CRS method. Since there were multiple random selection procedures involved in the CRS method, multiple simplified SNP combination sets can distinguish all distinct varieties in Train_Data in theory. Then the CRS method was applied five times to obtain five simplified SNP combination sets without duplicating SNP.

#### Identification ability comparison of multiple sets of SNP combination

Uniting multiple sets of SNP combinations can improve independent identification reliability and fault tolerance. Still, on the other hand, to save the identification cost as much as possible, it is necessary to determine the optimal number set of SNP combinations. It should contain a relatively small size of markers and can well distinguish EDV and non-EDV in practical application.

Compared with the 404 k SNPs, the number of selected SNPs with CRS has been dramatically reduced. There would result in a high false-positive if we still use IS = 97.5% as the threshold to judge whether the variety in Train_Data was EDV or not. Testing multiple IS value levels is necessary to determine the optimal IS threshold for selected SNPs in EDV and non-EDV identification. In this study, one to four SNP combination sets from the above five simplified SNP combination sets were selected to form a union SNP combination set. The threshold of IS 95.5% to 99.5% by the step of 1% was set to identify EDV for the optimal threshold of IS determination. At each threshold of IS, all varieties can be partitioned into four categories, which are quantified as the numbers of true positives (TP), true negatives (TN), false positives (FP), or false negatives (FN). By comparing the results with EDV identified with 404 k SNPs, the parameters Precision (PR) (formula (1)), Recall (RC) (formula (2)), and F-score (F1) (formula (3)) [29] were used to comprehensively determine the most suitable union SNP combination set for varieties identification.

3 $$PR = \frac{TP}{{TP + FP}}$$

4 $$RC = \frac{TP}{{TP + FN}}$$

5 $$F1 = 2\frac{PR \cdot RC}{{PR + RC}}$$

Meanwhile, the reference methods Random Selection (randomly select SNPs from Train_Data, denoted as RS) and high PIC selection (randomly select SNPs with PIC more than 0.4 from Train_Data, denoted as HPS) were also applicated for results comparison. After that, we calculated the optimal union SNP combination's PIC value and their distribution on the chromosome with R language.

#### Independent testing of variety identification

To test the varieties' identification ability of the three methods CRS, RS, and HPS, with the optimal IS threshold obtained from the above analysis, we separately used the optimal union SNP combination set, the same amount of SNPs selected by RS or HPS, for independent datasets' EDV identification. Then by comparing these results with EDV and non-EDV discriminated with 404 k SNPs under the threshold of IS = 97.5%, the PR, RC, and F1 of CRS, RS, and HPS methods were calculated. The RS and HPS methods were repeated applicated five times, and the highest PR, RC, and F1 of them were kept for comparing with that of the CRS. Test_Data1 and Test_Data2 were separately used for independent external varieties and internal varieties identification.

#### Data availability

The CRS method was compiled with the R language and could be downloaded free on Github (https://github.com/KnessKness/Li_1).

#### Generation of 2D barcode

We used an online tool (available at www.barcode-generator.org) [13] to generate the 2D barcode for each distinct variety (non-EDV) in Train_Data with the most suitable union SNP combination set obtained in 2.3.3 part. The genotype-based on the SNP barcode was entered, and the 2D barcode was automatically generated. Once the barcode was scanned, it confirmed the information used for creating the 2D barcode.

## Results

### Characteristics of SNPs in Train_Data

**Fig. 4:**
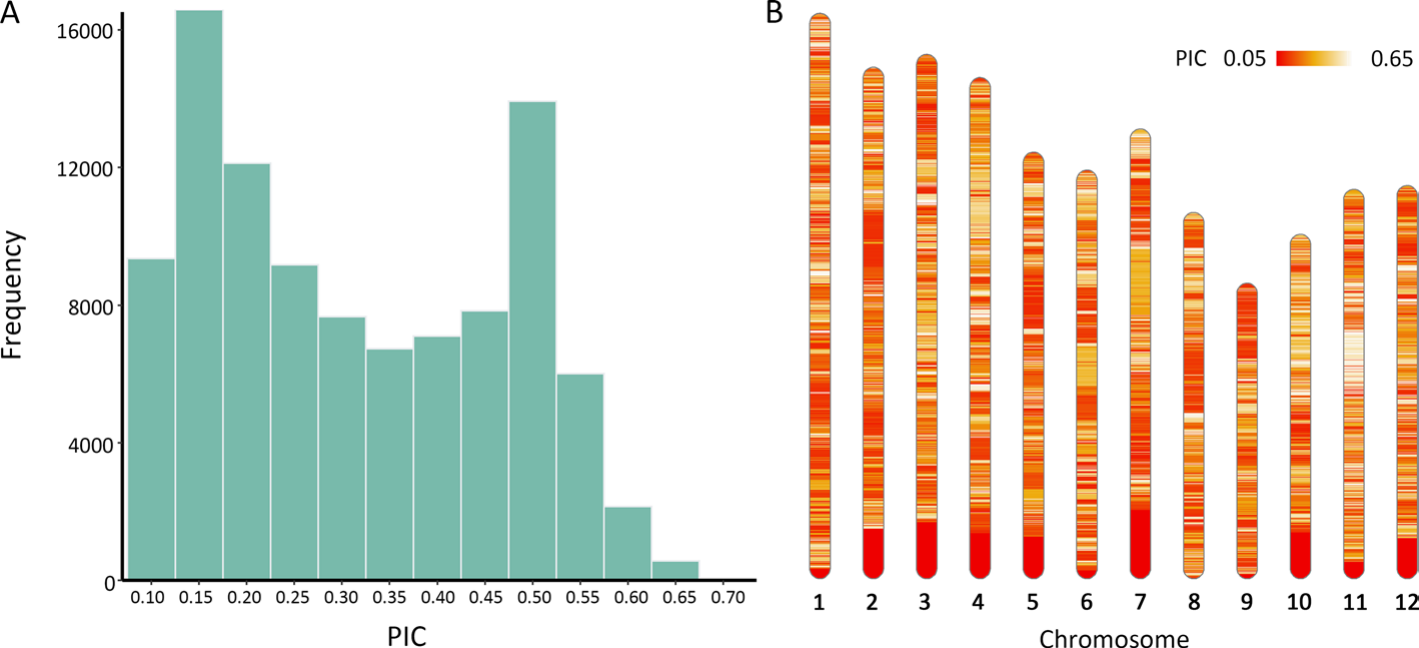
The PIC value of cSNPs in Train_Data and their distribution on chromosomes. **A** The histogram of PIC value of cSNPs in Train_Data; **B** The chromosome distribution of cSNPs in Train_Data

The 99, 253 cSNPs in the Train_Data were uniformly distributed on 12 chromosomes (Fig. 4B). The PIC value of these cSNPs ranged from 0.096 to 0.652. The frequency of most intervals of PIC of cSNPs was similar. The frequency of intervals of 0.10 < PIC < 0.15 and 0.45 < PIC < 0.50 were relatively higher than that of other intervals (Fig. 4A). Genomic similarity comparisons of varieties in Train_Data showed that the IS of each two varieties among 2629 varieties were less than 97.5%, with 404 k core SNPs in Train_Data. These 2629 varieties were deduced as non-EDV and were used for further varieties distinction in Train_Data.

### Training set modeling

**Fig. 5:**
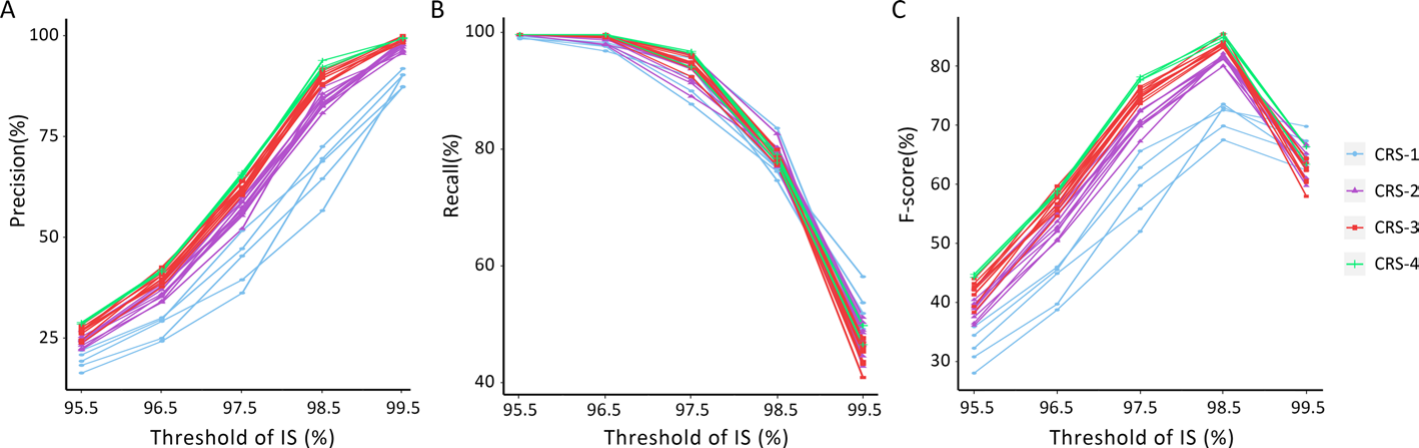
The performance of united SNP combinations screened out from CRS on EDV determination in Train_Data. **A**–**C** Separately represents the precision, recall and F-score of united SNP combinations screen out from CRS on EDV determination in Train_Data at different IS threshold. *Note* Legend refers to the Method-the number of simplified SNP combination set. For example, CRS-2 denoted as the union of two SNP combination sets that screened out from CRS method. Full combination of one to four combination sets from five simplified SNP combination sets were displayed. The detailed information of the united SNP combination sets was listed in Additional file 1: Table S1

By application of the CRS method five times, at last, we obtained five simplified SNP combination sets separately involving 118, 136, 121, 116, and 136 SNPs, without any duplicate SNP among these SNP combination sets. In order to improve the generalization and robustness of a single SNP combination set, one to four simplified SNP combination sets were united to determine EDV with different IS levels (Fig. 5). The EDV determination results showed that the PR increased as the threshold of IS increased (Fig. 5A), while it was the opposite for RC (Fig. 5B). The more simplified SNP combination sets involved, the higher precision was observed at the same level of the IS threshold. However, this was not true for RC. F-score is a measure of a model's accuracy on a dataset, which is denoted as the harmonic mean of PR and RC. Our results showed that when the threshold of IS = 98.5%, the union of three simplified SNP combination sets (118 + 136 + 136 = 390) has the best performance with F-score 85.4% (Fig. 5C), precision 91.7%, and recall 80.0%. Thus, the optimal IS threshold for the optimal united SNP combinations (390 SNPs) was determined as 98.5%.

### EDV and non-EDV determination performance with CRS, RS, and HPS methods

**Fig. 6:**
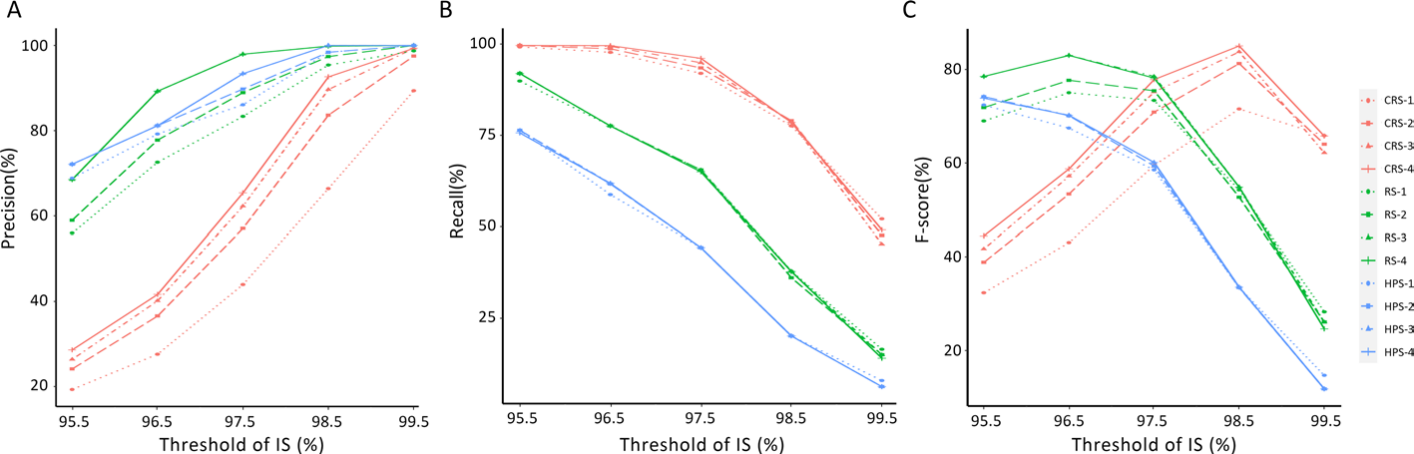
The average performance of united SNP combination sets screened out from CRS, HPS and RS on EDV determination in Train_Data. **A**–**C** Separately represents the average precision, recall and F-score of united SNP combinations screen out from the three marker selecting methods on EDV determination in Train_Data at different IS threshold. *Note* Legend refers to the Method-the number of simplified SNP combination set, as described in Fig. 3

EDV determination performance with CRS, RS, and HPS methods is shown in Fig. 6. As the number of SNP combination set increased, the precision of EDV determination increased in all three methods, while there was no significant difference in the recall of EDV determination. As the threshold of IS increased, the overall precision of EDV determination increased, and the recall decreased as a whole in all the three methods. When the IS threshold was less than 98%, the precision of the HPS and RS method were higher than that of the CRS method, while IS threshold was more than 98.5%, there was no significant difference in the precision of the three methods (Fig. 6A). The recall of the CRS method was higher than that of the other two methods (Fig. 6B). F-score showed that at the threshold of IS = 98.5% and 99.5%, the performance of CRS was better than HPS and RS. Especially at the threshold of IS = 98.5%, the average F-score of CRS with three and four SNP combination sets separately were 83.2% and 82%, which showed very similar good EDV determination performance in this study. Moreover, the union of three simplified SNP combination sets (118 + 136 + 136 = 390) has the best performance with F-score (Fig. 6C) as described in the above part.

**Fig. 7:**
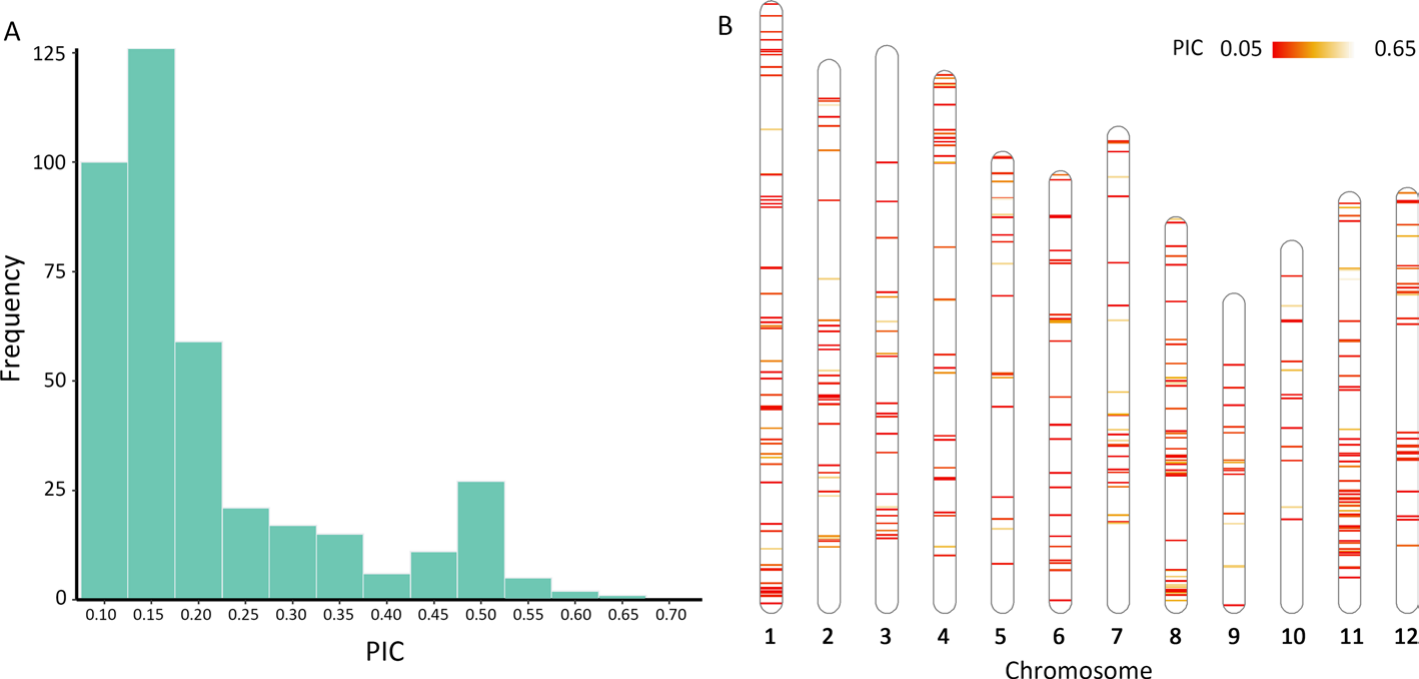
The PIC value of the 390 SNPs screened out with CRS and their distribution on chromosomes. **A** The histogram of PIC value of 390 SNPs screened out with CRS; **B** The chromosome distribution of 390 SNPs screened out with CRS

Meanwhile, the PIC value of the 390 SNPs with the best performance in EDV determination selected with the CRS method ranged from 0.096 to 0.652. The 390 SNPs were distributed on 12 chromosomes, and most of them with relatively low PIC value (Fig. 7), less than 0.2. Our results showed that the SNP combination set with high polymorphic information (PIC) of individual SNP was not necessarily better at EDV-discrimination than that with low polymorphic information (PIC) of some SNPs. This is possible due to the redundancy among these high PIC SNPs. With the same number of SNPs (390) selected with HPS or RS methods, all non-EDV in Train_Data also can be distinguished from each other. However, when the number of SNPs decreased, the CRS method showed significantly better performance on non-EDV discrimination than HPS and RS methods (data not shown).

At last, the SNPs of the union of three simplified SNP combination sets (118 + 136 + 136 = 390) were selected for rice fingerprint map construction of 3024 varieties in Train_Data. The united SNP combination sets of 3024 varieties were listed in Additional file 2: Table S2. Furthermore, Additional file 3: Table S3 lists the metadata information for the 3 K RG accessions. The 2D-barcode of each variety can be obtained with the unique ID of variety in the 3 K rice genome project and the 390 SNPs information with online software (available at www.barcode-generator.org).

### The detecting power of external variety identification

**Fig. 8:**
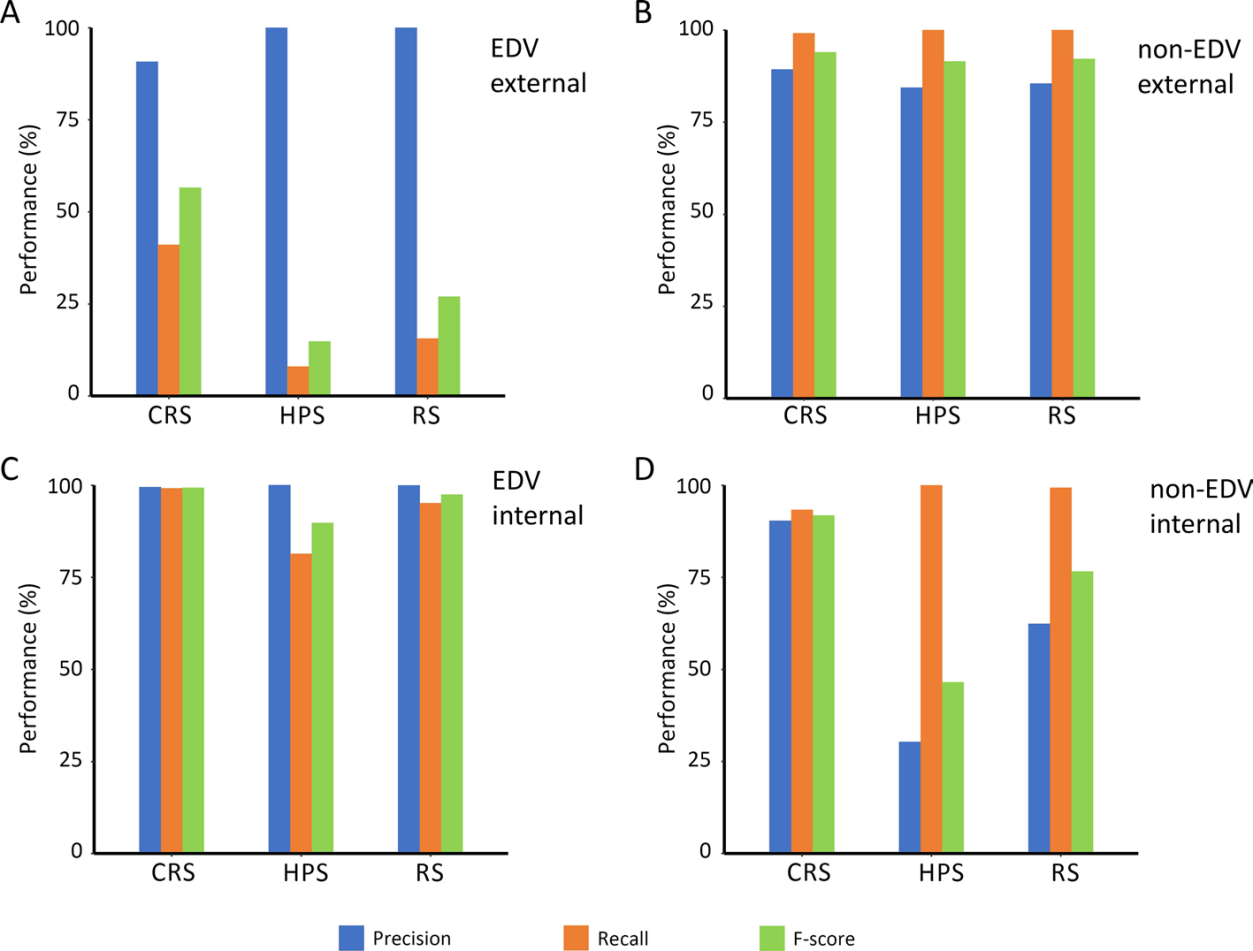
The performance of 390 SNPs screened out from CRS, RS and HPS on variety identification in Testing datasets. **A**, **B** Separately represents EDV and non-EDV identification performance in Test_Data1; **C**, **D** separately represents EDV and non-EDV identification performance in Test_Data2

By comparing the varieties in Test_Data1 and Train_Data with 404 k SNPs, there were 263 varieties in Test_Data1 determined as EDV of the varieties in Train_Data at the threshold of IS = 97.5%. While with the 390 SNPs selected with the CRS method, 129 varieties were determined as EDV at the threshold of IS = 98.5%. The precision and recall of the EDV determination with the CRS method were 83.72% and 41.06%, respectively. While there were 21 and 41 EDVs determined by HPS and RS method, respectively. The order of the F-score of the three methods is CRS (56.5%) > RS (27.0%) > HPS (14.8%) (Fig. 8A). For the 1305 non-EDVs in the Train_Data1, the three methods showed similar precision, recall, and F-score, and the CRS method showed relatively better performance (Fig. 8B). These results indicated CRS method was better than HPS and RS method in independent external varieties identification.

### The detecting power of internal variety identification

By comparing the varieties in Test_Data2 and Train_Data, with 404 k SNPs, there were 371 varieties among 401 in Test_Data2 determined as EDV to the varieties in Train_Data at the threshold of IS = 97.5%. While with the 390 SNPs selected with the CRS method, 368 varieties were determined as EDV at the threshold of IS = 98.5%. Only 302 and 353 EDVs were determined by HPS and RS method, respectively. And the order of the precision, recall and F-score of the three methods were HPS (100%) > RS (99.9%) > CRS (99.5%), CRS (99.2%) > RS (95.2%) > HPS (81.4%), CRS (99.3%) > RS (97.5%) > HPS (89.8%), respectively (Fig. 8C). For the 30 non-EDVs in the Train_Data2 determined with 404 k SNPs, the CRS method has higher precision and F-score than HPS and RS. The order of the F-score of the three methods was CRS (91.8%) > RS (76.6%) > HP (46.5%) (Fig. 8D). These results suggested that the CRS method was significantly better than the other two methods in identifying independent internal varieties.

## Discussion

A rapid and effective crop variety identification method should meet the high-resolution requirements of distinct variety discrimination, easy operation, standardization, and automation. Because most SNPs' genotype is bi-allelic (inbreeding lines) or tri-allelic (including hybrid lines), in theory, if there were n distinct varieties in the training dataset, at least p (2p = n or 3p = n) makers needed for distinguishing all varieties. For example, the combination of at least ten bi-allelic SNPs can discriminate 3024 rice varieties in the ideal situation. However, because of the enormous SNP markers contained in the genome sequencing data (m), selecting p markers from m is too large to obtain the optimal SNP combination set. Except for this big challenge, accurate identifying varieties in independent tests is another crucial challenge for crop variety identification[30]. It is well known that DNA fingerprinting has been applied for cultivar identification in various crop species, including cereals, vegetables, fruits, oilseeds, and nuts [1, 28]. However, strictly speaking, these techniques were not used for “identification” but for “distinction of a limited number of cultivars,” since, in most cases, they were applied for a limited number of definitive cultivars [28]. Even some strategies cannot distinguish all varieties in certain studies due to the close relationship among the non-distinguished accessions and/or the limitation of identification methods. Only a few researchers recently developed specific Indel or target-SNP for successfully new varieties identification in soybean [31] and cucumber [22].

With the development and wide application of machine learning methods [32, 33], several scientists recently interested in combining near-infrared hyperspectral imaging and deep learning methods (such as convolutional neural network (CNN), Residual Network (ResNet)) to identify crop varieties. Because of that, different varieties of crop seeds have different characters and values [34–36]. Zhou et al. proposed a novel convolutional neural network-based feature selector (CNN-FS) for wheat variety identification with a large spectral dataset of more than 140,000 wheat kernels in 30 wheat varieties [34], and this method achieved a high accuracy (93.01%) and kept high precision (90.02%) with 60-channel features. Although deep learning methods showed powerful prediction ability in variety identification with seed near-infrared hyperspectral images, they may not be suitable for variety identification with genotype data at the present stage since each variety (class) has only one sample in the training set. Maybe these methods could be used in genotype data for variety identification soon when the cost of sequencing is greatly reduced.

In this study, we proposed the Conditional Random Selection (CRS) method for effectively identifying rice variety. This method screened out a few SNPs evenly distributed in the coding region of the whole genome of rice varieties in the 3 K rice project with the strategy of "divide and conquer" could discriminate all distinct varieties successfully, which greatly save the cost of variety distinction in the Training set. Compared to the SNP located in the non-coding region, the SNP in the coding region (cSNP) are more stable and trend related to genes and traits of crops, which will facilitate the varieties discrimination [37]. Meanwhile, we coded the method with R language to reduce the manual operation and realize identification automation. In practical application, with the optimal threshold of IS searched in this study, the SNP combination selected from the CRS method also showed sound performance on EDV and non-EDV identification in independent testing datasets.

There were three advantages of the CRS method compared to traditional methods. First, we chose a SNP combination set that can distinguish all training set samples as much as possible. Previous studies often kept several high polymorphic SNPs and then clustered the samples to check the resolution of these SNPs. If some samples could not be distinguished, then find out those SNPs with the highest resolution for particular sample pairs and add them to the original list of SNPs to improve the resolution [10]. However, redundancy may exist among these high PIC SNPs. In this study, the CRS method with the strategy of "divide and conquer" first randomly selected a few markers to distinguish a small number of samples several times, kept the highest resolution SNP combination among them, and then selected markers from the remaining markers to distinguish other samples several times, kept the highest resolution SNP combination and added it to the previous SNP combination, step by step, until all samples were distinguished. The selected SNP combination was then de-redundant to obtain a simplified marker combination that can distinguish samples as much as possible quickly. With R code written by ourselves, the discrimination of 2629 non-EDVs in Train_Data was finished within 3.5 h (Compute parameters: x86_64 CPU, 12-core Intel® Xeon® Processor E5-2650 v4, 30 MB Intel® Smart Cache, 2.20 GHz).

Second, by uniting multiple sets of simplified SNP combinations and searching the optimal threshold of IS, the set with a suitable number of SNPs showed sound performance on variety identification (EDVs and non-EDVs determination) in the training set. IS of SNP on the whole genome was a good index for estimating the genomic similarity [22]. Compared to the 404 k SNPs, the SNP in the simplified marker combination was greatly reduced. In order to grant the accuracy of variety identification with the small amount of SNPs, among 20 sets of united SNP combinations, the set with 390 SNPs (three sets of simplified SNP combinations united together) that screened out with the CRS method showed the best EDVs and non-EDVs determination at the threshold of IS = 98.5% than that of other sets. It also outperformed HPS or RS method when with the same number of SNPs (390). This further confirmed the superior practicability of the CRS method on variety identification in the Training dataset.

Third, the SNPs selected with the CRS method showed good performance on variety identification in the testing set. As well-known as that, the constancy of a variety is provided by a set of genes specific to it, while variations of genes make the variety is different from generation to generation. In other words, the constancy of a variety is not stable. Thus, the SNP combinations even performed well on distinguishing EDV and non-EDV in the Training dataset but not necessarily well on identifying EDV and non-EDV in the testing set. With the CRS method, we initially retained the polymorphic markers in EDVs, then screened out the optimal SNP combination to distinguish non-EDVs in the training set. This strategy possibly significantly reduced the probability of mis-determination on EDVs and non-EDVs in the testing set.

Although CRS methods showed well performance on variety identification in both Training and Testing datasets, it still could not grant 100% of identification accuracy in the testing set due to the limitation of sample size in the training set. As more and more varieties are developed, the need to properly identify and categorize them increases. Besides that, according to the International Union for the Protection of New Varieties of Plants (UPOV) convention, a variety is deemed to be essentially derived from an initial variety if it is (1) predominantly derived and (2) clearly distinguishable from the initial variety and (3) genetically conform to the initial variety (UPOV1991) [38]. Although in this study, we through the genomic similarity to judge a variety whether it is an EDV, this was only conforming to the third rule. The accurate EDV identification is better to be further determined in combination with other information (etc., morphological traits in field trail) in the application.

## Conclusion

In this study, we developed an effective variety identification method. We first selected a minimal set of SNPs to discriminate non-EDVs in the 3000 Rice Genome Project, then united several sets of simplified SNP combinations to improve its generalization ability for EDV and non-EDV identification in testing datasets. The method outperformed traditional feature selection methods. Furthermore, it provides a new way to screen out core SNP loci from the whole genome for DNA fingerprinting of crop varieties and be useful for crop breeding.

## Supplementary Information

**Additional file 1: Table S1.** The detail information of united SNP combinations.

**Additional file 2: Table S2.** The information of the united SNP combination sets of 3024 varieties.

**Additional file 3: Table S3.** Metadata information for the 3K RG accessions.

## Data Availability

All authors declare that materials described in the manuscript, including all relevant raw data, will be freely available to any scientist wishing to use them for non-commercial purposes without breaching participant confidentiality.

## Abbreviations

EDV: Essentially derived variety
PIC: Polymorphic information content
CRS: Conditional random selection
RS: Random selection
HPS: High PIC selection
PR: Precision
RC: Recall
F1: F-score
UPOV: Union for the Protection of New Varieties of Plants
